# Analysis of question text properties for equality monitoring

**DOI:** 10.1007/s40037-018-0478-x

**Published:** 2018-10-23

**Authors:** Daniel Zahra, Steven A. Burr

**Affiliations:** Peninsula Medical and Dental Schools, Faculty of Medicine and Dentistry, University of Plymouth, Plymouth, UK

**Keywords:** Language complexity, Equality and diversity, Assessment questions

## Abstract

**Introduction:**

Ongoing monitoring of cohort demographic variation is an essential part of quality assurance in medical education assessments, yet the methods employed to explore possible underlying causes of demographic variation in performance are limited. Focussing on properties of the vignette text in single-best-answer multiple-choice questions (MCQs), we explore here the viability of conducting analyses of text properties and their relationship to candidate performance. We suggest that such analyses could become routine parts of assessment evaluation and provide an additional, equality-based measure of an assessment’s quality and fairness.

**Methods:**

We describe how a corpus of vignettes can be compiled, followed by examples of using Microsoft Word’s native readability statistics calculator and the koRpus text analysis package for the R statistical analysis environment for estimating the following properties of the question text: Flesch Reading Ease (FRE), Flesch-Kincaid Grade Level (Grade), word count, sentence count, and average words per sentence (WpS). We then provide examples of how these properties can be combined with equality and diversity variables, and the process automated to provide ongoing monitoring.

**Conclusions:**

Given the monitoring of demographic differences in assessment for assurance of equality, the ability to easily include textual analysis of question vignettes provides a useful tool for exploring possible causes of demographic variations in performance where they occur. It also provides another means of evaluating assessment quality and fairness with respect to demographic characteristics. Microsoft Word provides data comparable to the specialized koRpus package, suggesting routine use of word processing software for writing items and assessing their properties is viable with minimal burden, but that automation for ongoing monitoring also provides an additional means of standardizing MCQ assessment items, and eliminating or controlling textual variables as a possible contributor to differential attainment between subgroups.

## Introduction

Many countries have enacted policy to protect from discrimination by demographic factors such as gender, ethnicity, and disability. Examples of such legislative protections include the UK Equality Act [1], the European Equal Treatment Directives [2], and the American Equality [3] and Civil Rights Acts [4] which aim to make discrimination based on such protected characteristics illegal. In the context of such legislation, ‘Equality Monitoring’ has evolved in many educational settings. Equality monitoring is concerned with the evaluation of quality and fairness in assessments, and performance variation as a function of demographic factors. Most commonly, but not exclusively, equality monitoring centres on gender, ethnicity, and reported disability and evaluation of differential attainment between subgroups within these characteristics in a given assessment.

Not only is it important to ensure assessments do not discriminate as a property of these characteristics, it is also important from an educational point of view to ensure that differences in performance between candidates are unbiased by these demographic variables. If they are, the assessment does not reflect the candidates’ true ability, but rather their ability plus or minus the effect of the interaction between the assessment tool and demography. This is a position realized and supported by professional bodies such as the British Medical Association and the American Medical Association [5, 6], who require ongoing monitoring of cohort and demographic variation.

This is a commendable first step, but medical schools are often reluctant to make such monitoring processes public. This is understandable in the current political climate of higher education, but it is nonetheless detrimental to furthering our understanding of how and when demographic variation affects assessment quality and fairness and how assessment tools can be refined to reduce such biases. Anecdotally, schools occasionally find demographic differences in assessment performance, but do not necessarily have any agreed upon way of exploring these differences further, or a willingness to search for the underlying causes. This may be in part due to a lack of methodologies that are easy to employ routinely. Improving the range of assessment properties which can be evaluated for demographic variation as either explanatory variables or excluded as such forms the focus of this paper. In particular we advance the argument that ‘demographic differences’ may not be demographic per se, but the result of the language properties of assessment items. We propose here a method of evaluating the relationship between the language complexity of assessment items, assessment performance, and demographic variables as an additional tool for conducting post-test analyses and evaluation of assessment quality and fairness beyond simple, descriptive, demographic analyses.

Although research has considered factors such as candidate ethnicity, gender, and disability [7, 8], the findings are often restricted to the reporting of mean differences in assessment scores. Occasionally there may be speculation as to the underlying causes, but without further investigation. This may be a result of the sensitivities related to protected characteristics, and the potential negative impact of research findings being misreported or presented out of context in the wider media. However, it is an important area that should be addressed if progress is to be made in eliminating causes of differential attainment that are external to the student and their ability, and which undermine the validity and reliability of assessment tools.

The question of how and what to investigate as underlying factors of differential attainment presents further barriers to this essential but sensitive research. In an effort to move the field beyond merely monitoring and describing differences, we outline here a means of evaluating the textual properties of question text from single-best-answer multiple-choice questions (MCQs). After outlining a method, we explore the viability of conducting analyses of text properties or language complexity, indexed by Flesch Reading Ease (FRE) [9], Flesch-Kincaid Grade Level (Grade) [10], word count, sentence count, and average words per sentence (WpS). Both FRE and Grade have been used to assess the complexity of written materials in a wide range of environments, including healthcare settings [11, 12]. We have adopted them here because of the range of commonly used software which can calculate them, and the relative reliability of measures such as word count in comparison to more complex assessment of syntactic features. However, we acknowledge their limitations with respect to the assumptions of the different linguistic models on which they are based [13, 14] and the variable reliability between different measures [15, 16]. The specifics of calculating our chosen measures are detailed in the Method section. Word count, sentence count, and WpS have been included to provide context to these other measures, and to explore how FRE and Grade may vary as a function of these contextual measures which are salient to assessors during question writing.

We also acknowledge the discussion and research concerning other sources of bias in assessments. For example, the arguments that questions on particular topics or subject areas such as male or female specific pathologies may bias an assessment in favour of one or another subgroup due to their familiarity with the content outside of the educational environment. Though these are interesting arguments and areas we would encourage researchers to continue to explore, the focus of our work here is on question structure and the linguistic properties. These are universal factors that apply regardless of content. As such, developing our understanding of their impact and how to monitor them should be of interest and value to work on content-related biases, either as a means of controlling for language complexity, or allowing the interactions between structure and content to be investigated.

Our aim is to present a practical means of conducting these analyses both at the question-writing stage, and continuously throughout the assessment process. We hope this will provide additional data, separate from but related to potential demographic differences, that can be used by assessors to develop their understanding of one possible cause of variation, question text complexity, or rule it out as an underlying cause. With this aim in mind, we operationalize assessment bias or inequality as systematic variation in performance across demographic subgroups as a function of language complexity. Each of these components is discussed in detail below.

## Method

We describe here how a corpus of vignettes can be compiled and analyzed using Microsoft Word’s (‘Word’) native readability statistics calculator and the koRpus text analysis package [17] for the R statistical analysis environment (‘R’) [18]. It should be noted that there are other packages available for R that are designed to conduct similar analyses, but koRpus has clear and comprehensive supporting documentation. R and user-created packages such as koRpus are available through the Comprehensive R Archive Network [19] under GNU General Public Licence. We provide an example of estimating the Flesch Reading Ease (FRE), Flesch-Kincaid Grade Level (Grade), word count, sentence count, and average words per sentence (WpS), and describe how these properties can be combined with equality and diversity variables. We have chosen to focus on MCQs as they are widely used in medical education assessments, and are frequently stored as an electronic database of question text amenable to the analysis techniques we propose.

### Sampling

The tools described here, Word and koRpus, both operate on blocks of text and as such it is necessary to compile all of the question text for each item in a given assessment. Being designed for word-processing individual documents or blocks of text within a single document, Word is suited to analysis of individual items as they are written, whereas koRpus can make use of basic programming in R to process multiple blocks of text stored in either separate documents or separate cells of a spreadsheet. This ability to automate the process may facilitate its adoption for routine analysis.

In addition to item text, demographic information from candidates sitting the assessment is required, as is their individual performance data in order to assess any relationships between text properties, demographic factors, and performance. Performance in this context is defined as individual candidate scores for each item, though as discussed later, the analysis might be extended to consider the impact of language complexity on response selection and other measures of item performance (e. g. discrimination or response rate).

For this discussion, the focus is on the language complexity of items in a 125-item, single-best-answer MCQ-based assessment sat in 2016 by 345 Bachelor of Medicine–Bachelor of Surgery candidates. The demographic profile of this group is shown in Tab. 1.

**Table 1:** Demographic profile of candidates

Demographic factors		Total
Gender	Female	171
	Male	174
Ethnicity	White	201
	Asian	95
	Other	39
	Missing^a^	10
Disability	No known disability	309
	Specific learning difficulty	17
	Other disability	19
Total sitting the assessment		345

^a^Excluded from subsequent analyses of Ethnicity

The assessment comprised 125 items. The vignettes from each of these were collated for coding and combination with candidate item-level performance data. In our example, no items were removed from the assessment, but it is important to include only items that contribute to the final candidate outcomes.

In summary, the data required for such analysis is the text from each MCQ vignette, individual candidate scores for each item, and demographic information for each candidate. Once collated, these provide a dataset with candidates’ demographic information, their scores for every item, and the vignette text of each item.

### Measures of language complexity

All vignettes were tokenized using the koRpus package for the R statistical computing environment. Tokenization is the process of dividing the block of text into meaningful units at an appropriate level for the intended analysis: words in this case. This package was then used to calculate Flesch Reading Ease and Flesch-Kincaid Grade Levels for each vignette.

Flesch Reading Ease (FRE) provides a value between 0–100, with higher values indicating the text is easier to read. The recommended FRE for most texts is between 60–70 [20]. It is calculated as FRE = 206.835 − (1.015 × ASL) − (84.6 × ASW), where ASL and ASW are average sentence length and average number of syllables per word.

Flesch-Kincaid Grade Levels (Grade) provides an estimated US Grade-School level for comprehension of the text. For example, a Grade of 6 indicates the typical 6^th^ Grader could understand the text (11–12 years of age). The recommended Grade for most texts is 7–8 [20]. It is calculated as Grade = (0.39 × ASL) + (11.8 × ASW) − 15.59.

The number of words, sentences, and average words per sentence were also calculated for each vignette using koRpus.

FRE and Grade were also calculated for each vignette in the example assessment corpus using Word 2016’s [21] in-build readability statistics function for comparison to the output from koRpus. Statistics are computed in Word as part of its grammar- and spell-checking functions (instructions for most recent versions of Word can be found at tinyurl.com/qey8xdb).

With respect to the cost implications of these methods, the R statistical environment and associated analysis packages are open-source [17–19], and Microsoft offers a range of licencing options of varying costs. There are also freely available open-source word-processing software packages such as OpenOffice (see openoffice.org) which can calculate similar readability statistics.

### Candidate performance data

In our school, test items are drawn from a bank of possible items, and each item has a unique question identifier (Bank#). These were stored alongside the vignette text for each item, and used to identify responses and scores for each item, for each candidate, in each test. Combining candidate performance data with demographic data allowed us to collate all scores for each demographic group to each test item. These were then averaged to provide average (mean) item scores for each item overall and by demographic subgroups, and link these average item scores to the language complexity measures of the vignettes for each item (FRE, Grade, Word Count, Sentence Count, and WpS).

### Design and analysis

The relationships between each of the language complexity measures (FRE, Grade, Word Count, Sentence Count, and WpS) and performance (average item score) were explored using correlational analyses to calculate Pearson correlation coefficients and associated *p*-values. These were conducted overall and for each of the demographic subgroups individually (Male and Female gender groups; White, Asian, and Other ethnicity groups; No known, specific learning difficulty, and other disability groups); *p*-values were not adjusted for multiple comparisons given the illustrative nature of this example. As will be seen from the results section, the conclusions would remain the same if adjustments were made.

### Comparison of koRpus and Microsoft Word 2016

The FRE values for the vignettes calculated by koRpus (*M* = 60.16, *SD* = 13.10, range = 59.84) and Microsoft Word (*M* = 51.73, *SD* = 14.71, range = 62.10) were positively correlated (*r*(125) = 0.75, *p* < 0.001). The Grade of the vignettes calculated by koRpus (*M* = 8.21, *SD* = 2.32, range = 12.74) and Microsoft Word (*M* = 9.59, *SD* = 2.65, range = 17.00) were also positively correlated (r(125) = 0.66, *p* < 0.001). Questions where there was most deviation included a larger proportion of numerical data and unit annotation. This may be an artefact of how readability statistics are calculated, but to the authors’ knowledge there are no comparisons of our measures’ reliability between text with varying degrees of numerical content. Given the high correlation between Word and koRpus estimates of complexity, subsequent analyses used measures derived from koRpus. As automation within R, using koRpus, allows the application of this approach to larger corpora of vignettes, analysis across multiple tests or academic years, and the flexibility of R for conducting other post-test analyses, koRpus and R would be our recommended statistical tools for such analyses.

### Worked example

For automated processing using R, all vignettes were compiled row-wise into a spreadsheet, and variables added to identify the academic year, test, item number, and Bank#. This allows easy identification of items by their position in a test, a given test, year, and bank identifiers should they need to be excluded from the analyses or their performance scrutinized more closely.

**Fig. 1:**
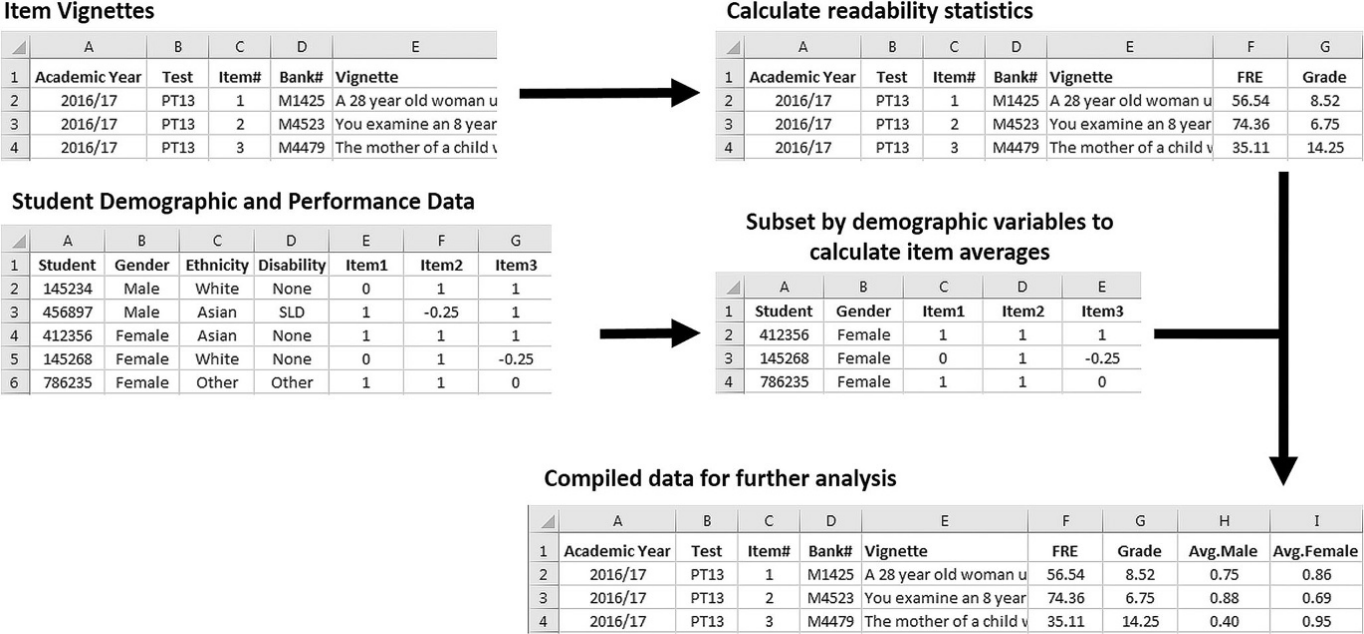
Data compilation process

koRpus was then used to tokenize each vignette sequentially, compute, and record to the spreadsheet the language complexity statistics of interest: FRE, Grade, Word Count, Sentence Count, and Words per Sentence. Once this information was compiled for each item, average item scores were derived using candidate scores. Having both Bank# and test item numbers allows the performance data to be subset by each item, then by candidate demographic information, and then for average performance to be calculated from this subset of scores and added to the language complexity data. This process is outlined in Fig. 1, and example R script is available from the authors on request.

**Fig. 2:**
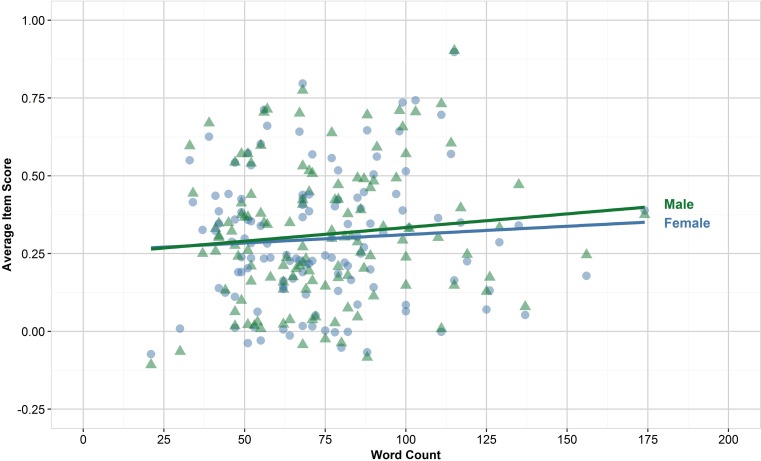
Scatterplot of word count by average item score. Points coloured by gender for illustration. Items were scored −0.25 for incorrect, 0 for don’t know, and 1 for correct

The correlation between each measure of language complexity and the average item scores are shown in Tab. 2. None of the correlations between measures of language complexity and performance were statistically significant either overall or for any of the demographic subgroups. It is, however, interesting that as FRE increased, so did average item scores, whereas as Grade increased, scores typically decreased. The correlation for Words is displayed in Fig. 2 for illustration, where the points have also been identified by gender to demonstrate possible uses in evaluating differential impact of language complexity; but no gender differences were found in this dataset.

**Table 2:** Pearson correlation coefficients between measures of language complexity and item scores, by demographic factor levels. No correlations were statistically significant at *p* = 0.05

		Measures				
Factors		FRE	Grade	Word count	Sentence count	WpS
Gender	Female	0.038	−0.029	0.069	−0.039	−0.018
	Male	0.074	−0.051	0.110	−0.026	0.005
Ethnicity	White	0.057	−0.037	0.080	−0.047	0.003
	Asian	0.062	−0.055	0.116	0.005	−0.021
	Other	0.047	−0.036	0.082	−0.032	−0.028
Disability	No known disability	0.060	−0.043	0.088	−0.033	−0.008
	Specific learning difficulty	0.055	−0.056	0.106	−0.012	−0.036
	Other disability	0.008	0.005	0.085	−0.039	0.049
Overall		0.056	−0.043	0.094	−0.027	−0.015

*FRE* Flesch Reading Ease, *WpS* words per sentence

## Conclusions

Given the monitoring of demographic differences in assessment for assurance of quality and fairness, the ability to easily include analysis of question text complexity may be useful in exploring possible causes of differences if and when they occur. Microsoft Word provides data comparable to a specialized linguistic analysis tool, suggesting routine use of word processing software for writing items and assessing their properties is viable with minimal burden to the staff involved. This would allow question writers to include some degree of standardization of these language complexity factors when constructing MCQ assessments, eliminating or controlling one possible contributor to differential attainment between subgroups.

Our proposed method of language complexity analysis may also be used to develop an understanding of how, when, and to what extent properties of item vignettes affect performance, and this information may provide parameters for the standardization or even automatic generation of items [22]. This is a growing area of work and one that may usefully improve the fairness of knowledge assessments in medical education.

### Next steps

Having detailed a viable, easy to implement, and cost-effective means of evaluating the language complexity of vignettes, and its potential impact on student performance, wider implications such as thresholds for action and implementation in practice need to be considered. In order to provide a useful screening tool for identifying vignettes which may disadvantage a particular subgroup, more work is needed on how these measures of complexity relate to performance across different assessments, the magnitude of any effects, and their consistency across cohorts. It may also be valuable to explore the relationship between measures of language complexity and other measures of item performance besides average item scores; for example, item discrimination or response rates to each item. These measures in combination may provide a means of describing and evaluating the quality and fairness of assessments and assessment items that goes beyond the merely descriptive.

Though the method provides interesting information linking language complexity, average item scores, and student demographics, we would not encourage anyone who finds relationships between these factors to necessarily consider them causal in and of themselves; though they may point the direction for further exploration. Where institutions have existing methods of monitoring demographic variation in performance, this approach may provide a means of investigating what underlies any differences which do occur, or at the very least, ruling out language complexity as a factor in those differences.

## References

[1] Government Equalities Office (2010). Equality Act 2010.

[2] European Parliament (2006). Equal Treatment Directive 2006 (2006/54/EC).

[3] Merkley J (2017). Equality Act of 2017.

[4] Celler E (1964). Civil Rights Act of 1964.

[5] Lakhan SE (2003). Diversification of U.S. medical schools via affirmative action implementation. BMC Med Educ.

[6] Mathers J, Sitch A, Marsh JL, Parry J (2011). Widening access to medical education for under-represented socioeconomic groups: population based cross sectional analysis of UK data, 2002–6. BMJ.

[7] Woolf K, Rich A, Viney R, Needleman S, Griffin A (2016). Perceived causes of differential attainment in UK postgraduate medical training: a national qualitative study. BMJ.

[8] Flesch R (1948). A new readability yardstick. J Appl Psychol.

[9] Kincaid JP, Fishburne RP, Rogers RL, Chissom BS, Millington TN (1975). Derivation of new readability formulas (Automated Readability Index, Fog Count and Flesch Reading Ease Formula) for Navy enlisted personnel. Naval Technical Training.

[10] Ferguson E, James D (2002). Factors associated with success in medical school: systematic review of the literature. BMJ.

[11] Babarudeen S, Sabharwal S (2010). Assessing readability of patient education materials: current role in orthopaedics. Clin Orthop.

[12] Larson E, Foe G, Lally R (2015). Reading level and length of written research consent forms. Clin Trans Sci.

[13] Janan D, Wray D (2014). Reassessing the accuracy and use of readability formulae. Malaysian J Learn Instr.

[14] Crossley SA, Allen DB, McNamara DS (2011). Text readability and intuitive simplification: a comparison of readability formulas. Read Foreign Lang.

[15] Feng L, Jansche M, Huenerfauth M, Elhahad N (2010). A comparison of features for automatic readability assessment. Proceedings of the 23rd International Conference on Computational Linguistics: Posters.

[16] Mailloux SL, Johnson ME, Fisher DG, Pettibone TJ (1995). How reliable is computerized assessment of reliability?. Comput Nurs.

[17] Michalke M (2012). koRpus – ein R‑paket zur textanalyse. Paper presented at the Tagung experimentell arbeitender Psychologen (TeaP).

[18] R Development Core Team (2015). R: A language and environment for statistical computing.

[19] The Comprehensive R Archive Network. 2018. https://www.r-project.org/..

[20] Microsoft Corporation (2018). Test your document’s readability.

[21] Microsoft Corporation (2016). Microsoft Office Word 2016 MSO 16.0.4639.1000, 32-bit.

[22] Gierl MJ, Lai H, Turner SR (2012). Using automatic item generation to create multiple-choice test items. Med Educ.

